# The Hidden Threat: Maxillary Hardware-Associated Actinomycosis in Diabetes

**DOI:** 10.7759/cureus.96264

**Published:** 2025-11-06

**Authors:** Nidha Shapoo, Noella Boma

**Affiliations:** 1 Internal Medicine, New York Medical College, Metropolitan Hospital Center, New York, USA

**Keywords:** actinomycosis cervicofacial, anterior maxilla, diabetes type 2, facial cellulitis, maxillary hardware

## Abstract

Actinomycosis is a chronic granulomatous infection caused by anaerobic Gram-positive Actinomyces species. While cervicofacial actinomycosis is the most common form, involvement of the maxilla is rare due to its rich vascular supply. Osteomyelitis of the maxilla, caused by actinomycosis, is an uncommon but serious condition that can lead to extensive local destruction and soft tissue involvement in patients with implanted hardware and underlying immunocompromised state. We present the case of a 46-year-old male with poorly controlled type 2 diabetes mellitus and a history of right maxillary hardware placement following a zygomatic arch fracture. He presented with acute right facial swelling, pain, and trismus. Imaging revealed signs of maxillary osteomyelitis and facial cellulitis. Cultures from the surgical debridement grew *Actinomyces odontolyticus*, *Streptococcus epidermidis*, *Abiotrophia defectiva*, and *Veillonella parvula*. The patient was treated with surgical removal of the infected hardware and a prolonged course of intravenous and oral antibiotics, resulting in full clinical recovery. This case highlights the importance of early recognition and aggressive management of actinomycosis-related osteomyelitis in high-risk patients. Poor dental hygiene, implanted hardware, and systemic immunocompromising conditions such as diabetes mellitus serve as key predisposing factors. Multidisciplinary management and long-term antimicrobial therapy are essential to successful outcomes.

## Introduction

Actinomycosis is a rare, chronic, and often misdiagnosed infection caused by Gram-positive Actinomyces species [1]. These bacteria are commensals of the oral cavity, gastrointestinal tract, and female genital tract, and they require mucosal disruption to become pathogenic. The disruption allows the bacteria to invade deeper submucosal tissues, where oxygen tension is lower, favoring anaerobic growth. Cervicofacial actinomycosis accounts for up to 60% of all cases and typically presents as a slowly progressive, indurated mass with draining sinus tracts. In rare instances, particularly in immunocompromised patients, it may present more aggressively with abscess formation, osteomyelitis, and facial cellulitis. Poor dental hygiene, oral trauma, dental extraction, and dental implants are essential predisposing factors [2-7].

Maxillary involvement is exceedingly rare, seen in only 0.5-9% of cases in the cervicofacial area. The abundant vascularity of the maxilla generally confers protection against chronic infection; however, it can lead to extensive local destruction if not treated early [8]. There are a few reports of maxillary actinomycosis in the literature [8-15]. We present a case of maxillary actinomycosis associated with implanted hardware, causing extensive bone destruction leading to osteomyelitis and facial cellulitis.

## Case presentation

**Figure 1:**
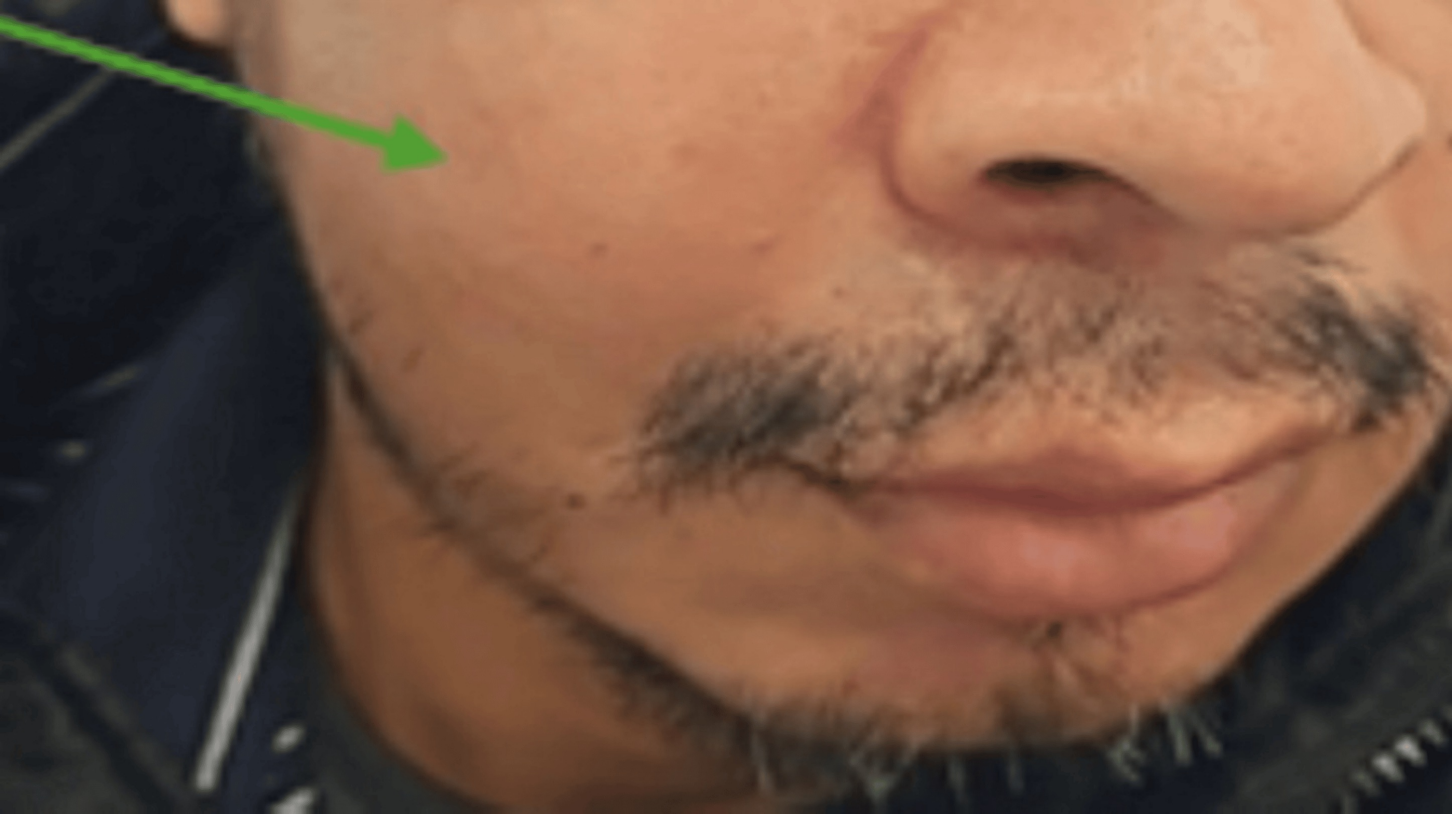
Right-sided facial swelling with erythema.

A 46-year-old Hispanic male, with a known history of poorly controlled type II diabetes mellitus (HbA1c 13.6%), presented to the emergency department with a two-day history of progressively worsening right-sided facial swelling, pain, erythema, and difficulty chewing (Figure 1).

He denied fever or chills. Notably, the patient had sustained a right-sided zygomatic arch fracture three months earlier, which was managed with open reduction and internal fixation of the right maxillary sinus, orbital wall, and nasal bone. Two months before presentation, he was treated for the right dental abscess with incision and drainage and a 10-day course of oral antibiotics. On examination, the patient had visible right facial swelling with overlying erythema, tenderness, and trismus. Oral examination revealed poor dental hygiene. Vitals were stable with a temperature of 37.2°C, heart rate of 90/min, and blood pressure of 140/88 mmHg. There were no signs of sepsis. The laboratory investigations revealed anemia, hyperglycemia, and elevated ESR and C-reactive protein, which are depicted in Table 1.

**Table 1:** Laboratory investigations upon admission.

Component (reference range)	Result
Hemoglobin (12-16 g/dL)	10.2 g/dL
Total leucocyte count (4000-7000/µL)	6200/µL
Blood glucose (74-109 mg/dL)	291 mg/dL
HbA1c (4-5.6%)	13.6%
ESR (0-20 mm/h), CRP (2-4 mg/dl)	40 mm/h, 80 mg/dl
Serum creatinine (0.6-1 mg/dL)	0.8 mg/dL
Blood urea nitrogen (6-24 mg/dL)	12 mg/dL
Serum sodium (135-145 mEq/L)	138 mEq/L
Serum potassium (3.5-5.2 mEq/L)	4.2 mEq/L
Serum ethanol <10 mg/dL	<10 mg/dL

**Figure 2:**
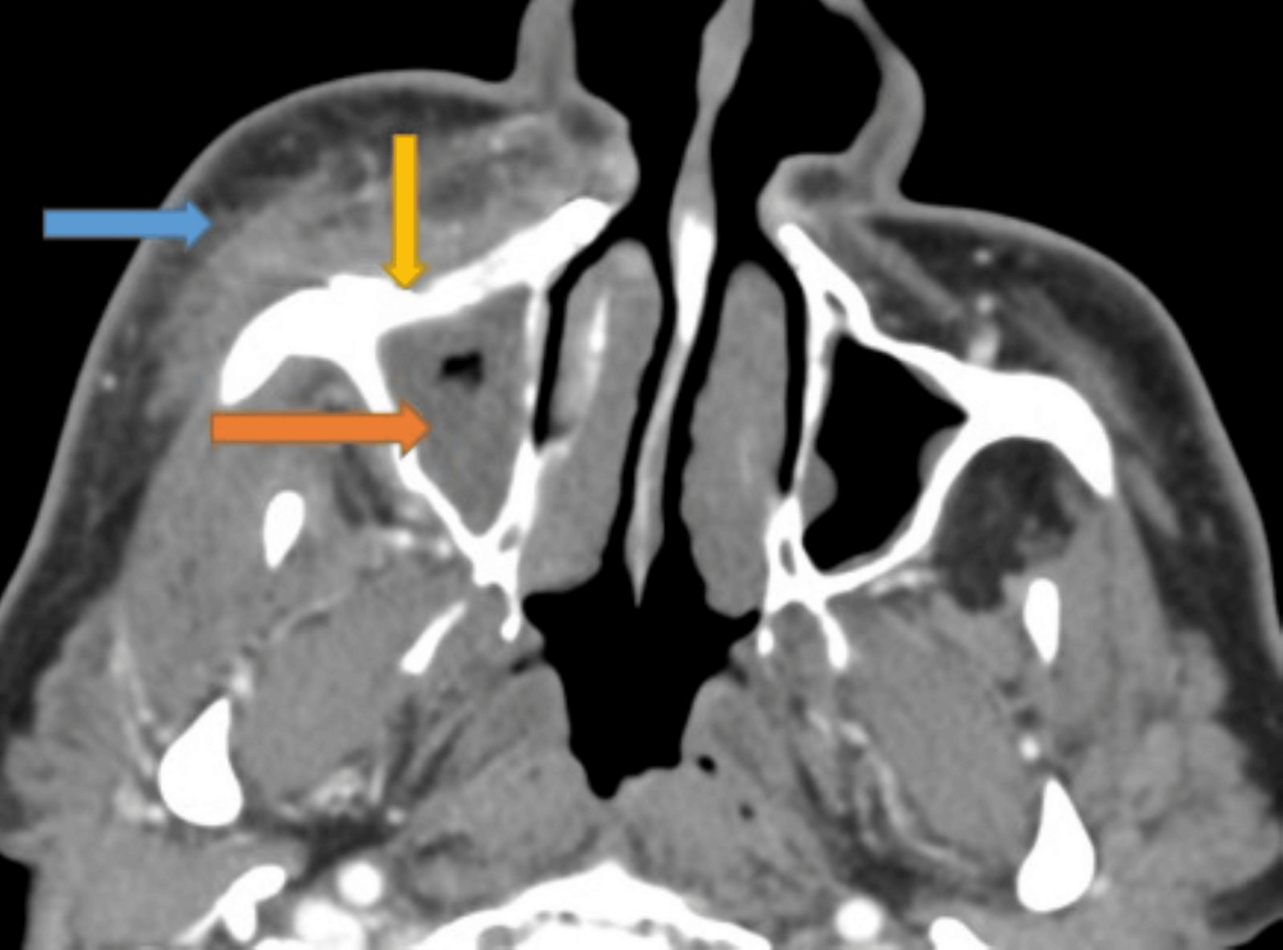
CT maxillofacial with contrast (sagittal view) showing the right maxillary hardware (yellow arrow), right maxillary sinus opacification (orange arrow), and right facial soft tissue swelling (blue arrow).

**Figure 3:**
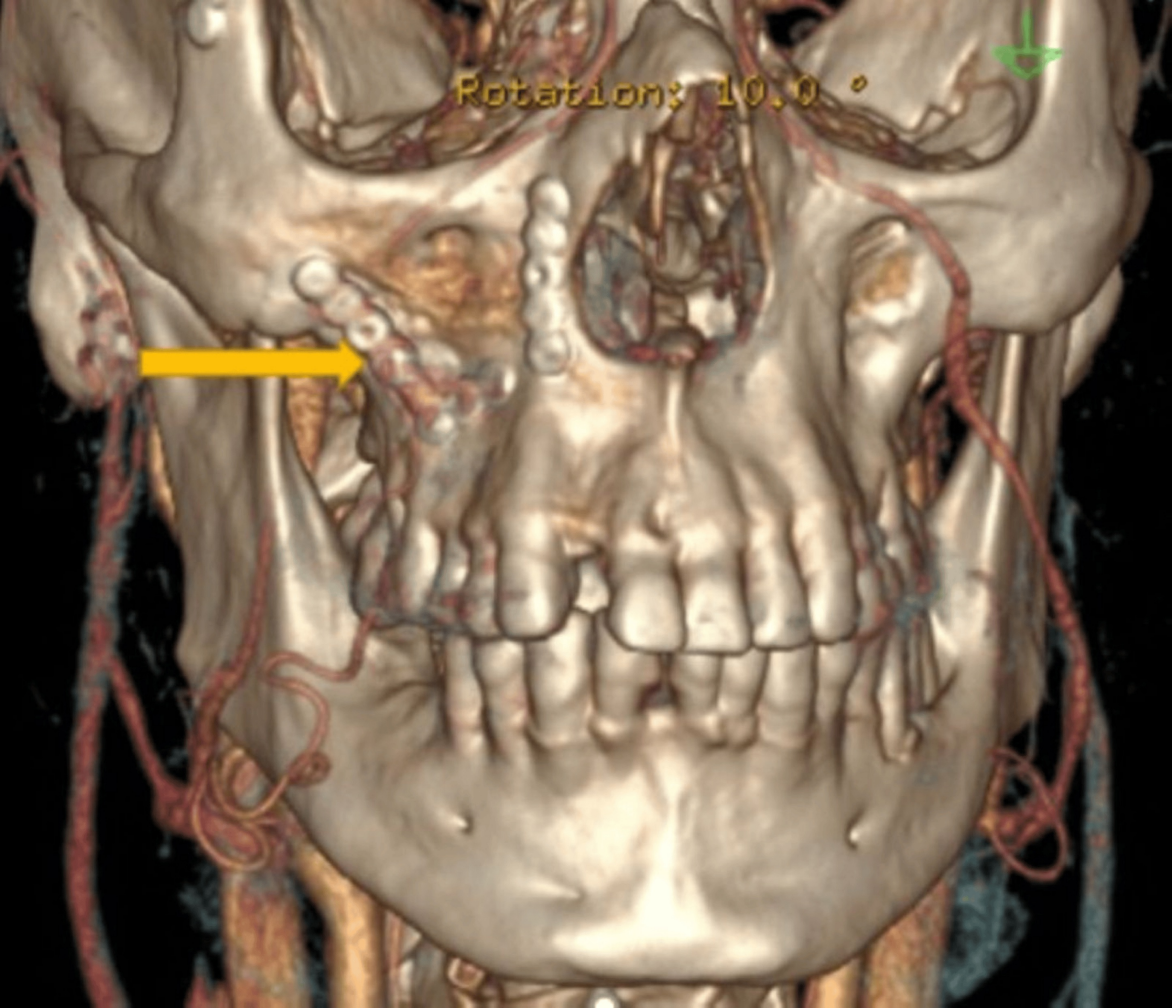
CT maxillofacial with contrast (3D view) showing right maxillary hardware (yellow arrow).

Computed tomography (CT) maxillofacial with contrast showed partial opacification of the right maxillary sinus with discontinuity of the posterolateral right maxillary sinus wall, bilateral facial cellulitis (right > left), and post-surgical changes and prior hardware in the right maxillary and orbital walls (Figures 2, 3).

**Figure 4:**
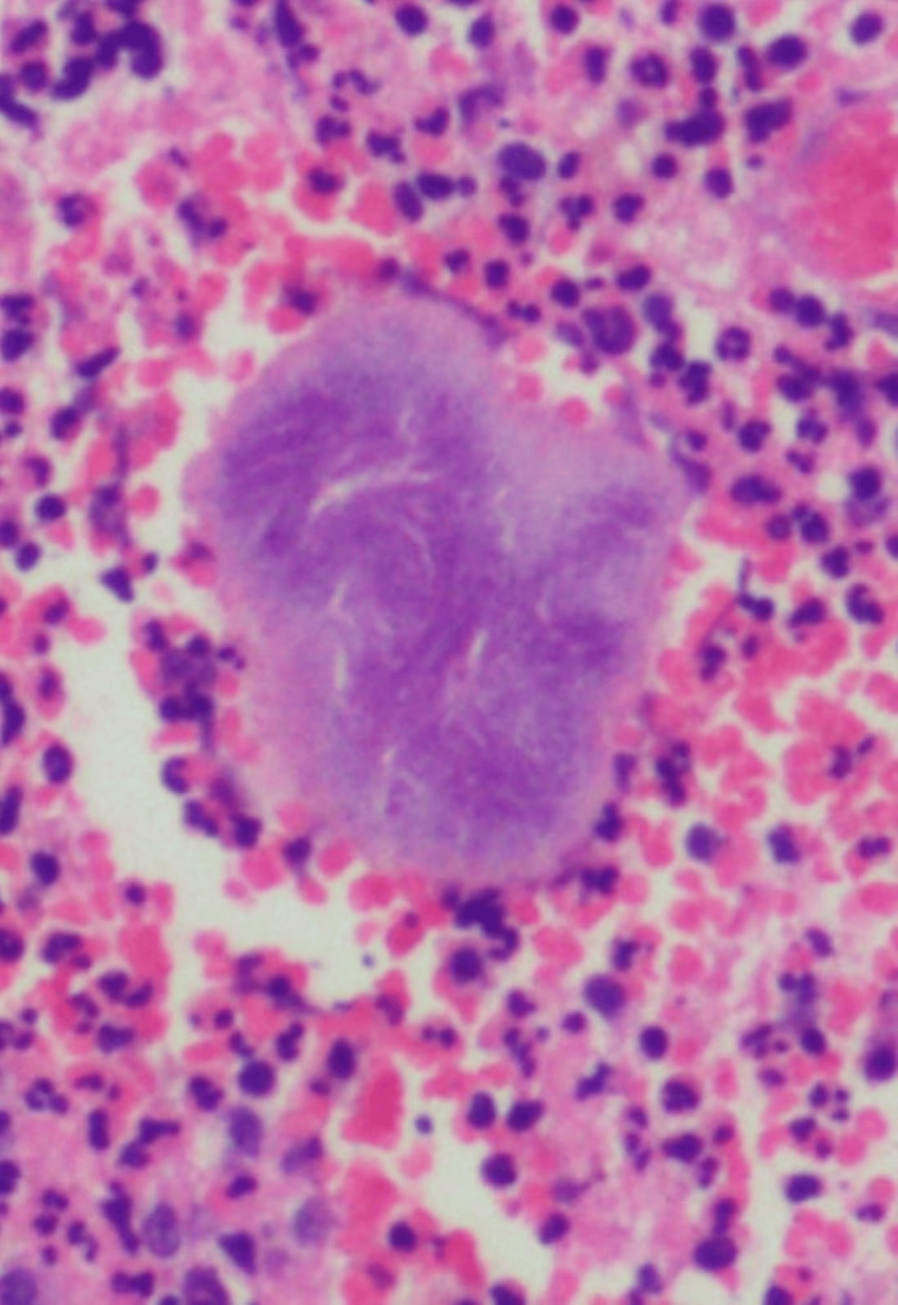
Histopathology shows characteristic sulfur granules within the abscess material (H&E stain, x400).

The patient was initiated on broad-spectrum intravenous antibiotics. Oral and maxillofacial surgery, general dentistry, and infectious disease were consulted. In view of recurrent infections and suspected osteomyelitis, surgical removal of the right maxillary hardware was performed. Intraoperative bone cultures grew *Actinomyces odontolyticus*, *Streptococcus epidermidis* (MSSE), *Abiotrophia defectiva*, and *Veillonella parvula*. Histopathology of the abscess material revealed sulfur granules (Figure 4).

The patient was treated with intravenous ceftriaxone 2 g daily with oral metronidazole 500 mg three times daily for a total of six weeks. Metronidazole was included to target anaerobes, such as *Veillonella*, and a prolonged regimen was chosen due to bone involvement and slow pathogen response. Upon discharge, oral amoxicillin-clavulanate (Augmentin) 875 mg twice a day was started with the plan to continue for six to 12 months. Blood glucose was stabilized using an inpatient insulin regimen. The patient demonstrated significant clinical improvement upon discharge. The laboratory results showed normalization of inflammatory markers and blood glucose. At his last follow-up, after three months of oral therapy, the patient had complete resolution of pain and swelling and remained asymptomatic. The repeat CT of the maxillofacial region showed complete resolution of the abscess. Augmentin was continued for another three months with monthly outpatient follow-up.

## Discussion

*Actinomyces israelii* is the most common pathogen isolated from the cervicofacial area; however, *Actinomyces odontolyticus* is more prominent in failed implants [3,7].

Sarkonen et al., in their study of 17 patients with 33 dental implant fixtures, found Actinomyces to be the most prevalent bacteria in these failed implants, colonizing 94% of the fixtures. *Actinomyces odontolyticus* was present in 84% of Actinomyces-positive fixtures, whereas *Actinomyces naeslundii* and *Actinomyces viscosus* were both detected in 32% and Actinomyces Israeli in 23% of implants [3].

Actinomycosis is often polymicrobial, with other anaerobes or facultative organisms such as *Streptococcus*, *Staphylococcus*, and *Veillonella* enhancing its pathogenicity [16]. The pathophysiology of actinomycosis osteomyelitis remains unclear; however, it is proposed that inflammation begins when the normal microbial flora is disrupted, leading to persistent inflammation and localized pathological alterations in the bone. The mandibular bone is more likely to develop osteomyelitis as compared to the maxilla due to relatively poor vascularization, leading to osteonecrosis [14].

In addition to local risk factors, systemic factors like diabetes mellitus, alcohol use, malnutrition, malignancies, HIV, solid organ transplantation, and biological agents, such as infliximab, are known to increase the risk of actinomycosis [9].

The presence of *Actinomyces odontolyticus* in our patient supports the fact that the infection originated from prior surgical manipulation, the presence of implanted hardware, and poor oral hygiene. The polymicrobial nature and the underlying poorly controlled diabetes mellitus further contributed to disease progression.

Diagnosis is challenging due to the indolent nature of the disease and its clinical mimicry of neoplastic or other chronic infectious conditions. Histopathology may reveal sulfur granules, and the diagnosis is best made by culture; however, <50% of cases are positive due to a prolonged incubation period and the requirement for anaerobic conditions [14]. Nucleic acid probes and polymerase chain reaction methods can be utilized for rapid identification, but are highly expensive [13]. CT imaging is essential in detecting bony involvement. The diagnosis in our case was confirmed by the growth of Actinomyces on bone culture with CT evidence of bone involvement.

Treatment involves extensive surgical debridement, removal of foreign material or necrotic tissue, and prolonged antibiotic therapy. No randomized controlled trials have assessed antibiotic protocols for cervicofacial actinomycosis. Most isolates exhibit susceptibility to beta-lactams, with the preferred treatment being an extended regimen of penicillin or its derivatives, accompanied by stringent glycemic management in diabetic patients. Due to the limited penetration of beta-lactams in bone, substantial intravenous dosages of amoxicillin or penicillin G must be administered first in severe instances. Acceptable options are clindamycin, macrolides (erythromycin, clarithromycin, or azithromycin), and doxycycline, noted for their superior bone penetration. Metronidazole is used because of the polymicrobial characteristics of the infection. Extended antibiotic regimens are necessary to achieve thorough eradication of Actinomyces and to avert recurrences [1]. The selection of antibiotics for our patient was contingent upon the antibiotic sensitivity of the pathogens, and ceftriaxone was selected for its simple daily dose regimen.

## Conclusions

Maxillary actinomycosis is an uncommon but serious complication of cervicofacial infections associated with implanted hardware, particularly in immunocompromised individuals. Clinicians should maintain a high index of suspicion in patients with risk factors, such as poor dentition, prior maxillofacial trauma or surgery, and uncontrolled diabetes. Prompt recognition, appropriate surgical debridement, removal of the failed hardware, and long-term antibiotic therapy are key to preventing complications and achieving full recovery.
